# 9-Vinylanthracene Based Fluorogens: Synthesis, Structure-Property Relationships and Applications

**DOI:** 10.3390/molecules22122148

**Published:** 2017-12-04

**Authors:** Mengjie Liu, Sawaros Onchaiya, Lewis Yi Fong Tan, Mohammad A. Haghighatbin, Tracey Luu, Tze Cin Owyong, Roozbeh Hushiarian, Conor F. Hogan, Trevor A. Smith, Yuning Hong

**Affiliations:** 1 Department of Chemistry and Physics, La Trobe Institute for Molecular Science, La Trobe University, Melbourne, VIC 3086, Australia; M.Liu4@latrobe.edu.au (M.L.); sawaros.onc@student.mahidol.ac.th (S.O.); 18768730@students.latrobe.edu.au (L.Y.F.T); M.Haghighatbin@latrobe.edu.au (M.A.H.); traceyl@student.unimelb.edu.au (T.L.); towyong@student.unimelb.edu.au (T.C.O.); R.Hushiarian@latrobe.edu.au (R.H.); C.Hogan@latrobe.edu.au (C.F.H.); 2 School of Chemistry, The University of Melbourne, Parkville, VIC 3010, Australia; trevoras@unimelb.edu.au

**Keywords:** aggregation-induced emission, anthracene, solvatochromism

## Abstract

Fluorescent dyes with aggregation-induced emission (AIE) properties exhibit intensified emission upon aggregation. They are promising candidates to study biomolecules and cellular changes in aqueous environments when aggregation formation occurs. Here, we report a group of 9-position functionalized anthracene derivatives that were conveniently synthesized by the palladium-catalyzed Heck reaction. Using fluorometric analyses, these dyes were confirmed to show AIE behavior upon forming aggregates at high concentrations, in viscous solvents, and when poorly solubilized. Their photophysical properties were then further correlated with their structural features, using density functional theory (DFT) calculation. Finally, we demonstrated their potential applications in monitoring pH changes, quantifying globular proteins, as well as cell imaging with confocal microscopy.

## 1. Introduction

The availability and proper design of fluorescent dyes are the fundamental elements in optical imaging, enabling highly specific and in vitro and non-invasive in vivo investigation of biomolecules [1]. Since they were first identified in the 19th century, the development of fluorescent dyes has been a popular research area and fluorescence imaging has become a valuable technique in both research and clinical settings [2,3,4]. The fluorescence of the conventional dyes can be markedly disabled when they self-aggregate, which is commonly known as aggregation-caused quenching (ACQ). This phenomenon is speculated to originate from the aromatic planar structures typically seen in these dyes, which causes strong π–π stacking interactions in aggregation and thus switch off the fluorescence [5]. Research groups have taken advantage of this ACQ effect in the detection of biomolecules. For example, Doria et al. selectively probed G-quadruplexes by using a naphthalene derivative, which remained aggregated and quenched in physiological conditions, but became disaggregated and emissive upon binding to the targets of interest [6]. However, conjugation of conventional dyes to biomolecules with multiple labeling sites can readily incur aggregation and therefore annihilate their emission [7]. To date, this is an issue that limits the usefulness of the conventional dyes in biomolecular applications.

**Figure 1:**
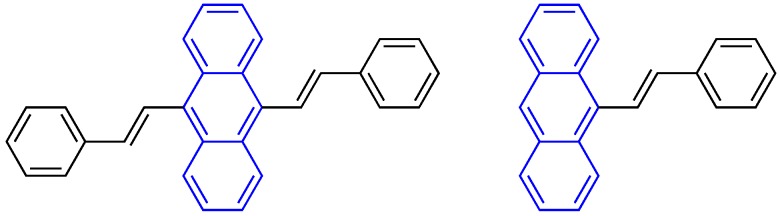
The structures of distyrylanthracene (**left**) and mono-functionalize anthracene (**right**).

Recently, a number of aggregation-induced emission (AIE) dyes have attracted increasing attention because they show no or weak emission in the solubilized state, but become progressively emissive when forming aggregates. Unlike “conventional” dyes, AIE-active dyes are typically non-planar but show a propeller shape, with a stator moiety surrounded by a number of aryl rotors. In a diluted solution, these rotors are able to freely rotate, thus rendering the dyes non-emissive by dissipating the energy through non-radiative pathways rather than fluorescence emission. In the aggregated state where the rotations are markedly constrained, the overall propeller shape prevents π–π stacking formation, and thus the dyes become emissive as the excess energy can now be released as light [5]. Symbolic examples of AIE-active dyes include a series of tetraphenylethene (TPE) derivatives comprised of an olefin stator surrounded by four phenyl rotators. They have been proved valuable in visualization of biomolecules and cellular environments such as mitochondrial phospholipids [8], nucleic acids [9], tumor proteins [10], and changes in intracellular pH [11], viscosity [12] and macromolecular crowding conditions [13]. The distyrylanthracene (DSA) derivatives are another set of typical AIE-active dyes (Figure 1 left). These molecules are structurally non-planar and therefore emissive in the aggregate state, owing to the steric hindrance induced by their bulky anthracene core. Several research groups have demonstrated the molecular stacking, crystal formation and photophysical properties of these dyes with various structural substitution [14,15,16,17], as well as their applications [18,19,20,21]. However, only a few studies have focused on the structural analogues, mono-functionalized anthracene derivatives (Figure 1 right), and their photophysical properties [22,23,24]. Additionally, their potential use in biological systems has yet to be demonstrated.

In this paper, we report a group of 9-position functionalized anthracene derivatives that were robustly prepared by the well-established palladium-catalyzed Heck reaction. We characterize their photophysical behaviors systematically to elucidate their structure-property relationships. Significantly, we were also able to demonstrate their potential applications in sensing pH, detecting proteins of interest, as well as cell imaging. 

## 2. Results and Discussion

### 2.1. Dye Synthesis

**Figure 2:**
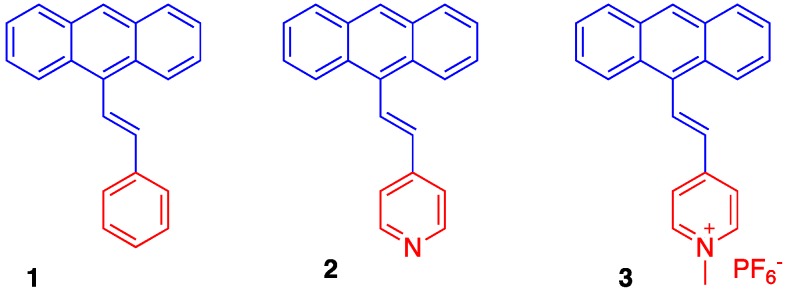
Chemical structures of dyes **1**–**3**.

The synthetic routes towards the desired 9-styrylanthracene-derived fluorophores (dyes **1**–**3**, Figure 2) and their characterization data are detailed in the Experimental section. In general, the core structures of these dyes could be conveniently synthesized using the palladium-catalyzed Heck coupling reaction, which linked the corresponding vinyl moieties to the anthracene system via a C=C double bond. Importantly, this reaction enabled synthesis of optically pure stereoisomers owing to its stereo-selectivity towards the *trans*-configuration. The synthesis of dye **1** was achieved by adapting the ligand-free Heck reaction strategy reported by Yao et al. [25], where the catalyst system consisted of Pd(OAc)_2_ and K_3_PO_4_ in dimethylacetamide (DMA) without any triaryl phosphine ligand. On the other hand, dye **2** was synthesized following the traditional Heck reaction strategy in the presence of the ligand PPh_3_ [22]. The positively charged dye **3** was simply prepared from dye **2** by refluxing in CH_3_I, followed by ion exchange in the presence of KPF_6_ as described in the Experimental section. All products were characterized by ^1^H- and ^13^C-NMR and mass spectrometry.

### 2.2. Structure-Property Relationships

#### 2.2.1. Absorption and Emission Spectra

**Figure 3:**
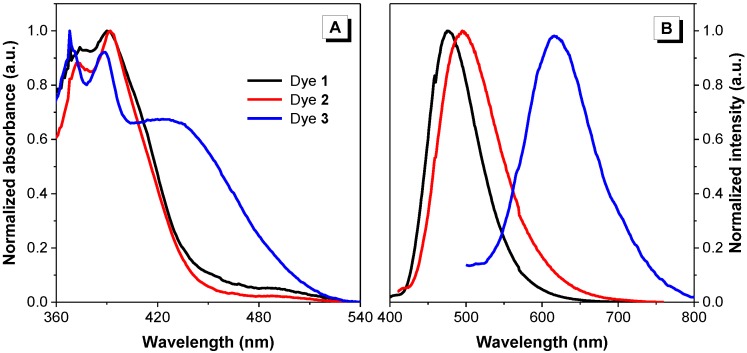
(**A**) Normalized absorption and (**B**) emission spectra in DMSO. Concentration = 10 µM. Excitation wavelength: 390 (1), 392 (2) and 420 nm (3).

As all dyes were found soluble in dimethyl sulfoxide (DMSO), their fluorescence properties were first measured in this solution. Their absorption profiles were analyzed by normalized absorption spectra (Figure 3A). Dyes **1** and **2** reached maximal excitation at very similar wavelengths (390 and 392 nm, respectively), while dye **3** exhibited a slight blue-shift and a second absorption maximum at about 420 nm. Subsequently, their emission spectra were measured after exciting the dyes at their recorded absorption maxima (Figure 3B). Dyes **1** and **2** exhibited emission maxima at 475 and 495 nm, respectively, with the absolute quantum yields of 0.60 and 0.03, respectively; however, dye **3** showed a marked red-shift with the maximum at 618 nm when excited at 420 nm with a quantum yield of lower than 1% in DMSO solution.

#### 2.2.2. Effect of Solvent Polarity

**Figure 4:**
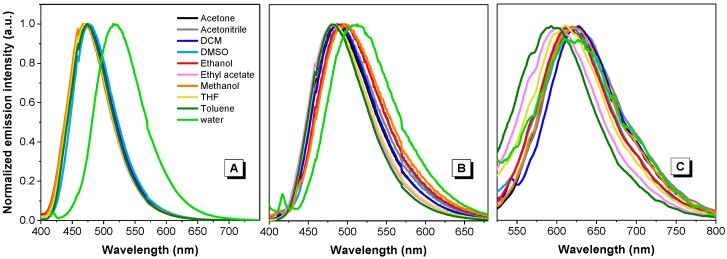
Normalized emission spectra of dyes **1** (**A**), **2** (**B**) and **3** (**C**) in different solvents. Concentration = 10 µM.

We analyzed the absorption and emission profiles of all the dyes in solvents with various polarity. The results are shown in Figure 4 and Figures S1–S3 in Supplementary Materials. The absorption maxima of dye **1**, obtained from the normalized absorption spectra, ranged from 385 nm to 395 nm in different solvents (Figure S1A), where water induced a small red-shift to 395 nm and a more pronounced shoulder at longer wavelengths. Compared with its emission spectra (Figure S1B), the strongest emission occurred in toluene, the most hydrophobic solvent used in this work. The lowest emission intensity was observed with water, a highly polar solvent. Compared with the normalized emission spectra shown in Figure 4A, the emission maxima in different solvents ranged from 470 to 475 nm; as an exception, water caused a marked red-shift to 515 nm.

The absorption maxima of dye **2** ranged from 387 nm to 392 nm in different solvents, where the absorption spectrum in water was markedly broader than those in other solvents (Figure S2A). Our subsequent experiments demonstrated that this dye formed nanoaggregates in water (ranged from 500–600 nm, discussed later) with greater size than the wavelength of incident light. This is expected to cause a Mie scattering effect, which broadens the absorption spectrum. Its emission was strongest in toluene and weakest in methanol (Figure S2B), where its emission maxima ranged from 480 nm to 500 nm. Similar to the case of dye **1**, water caused a slight red shift to 513 nm (Figure 4B).

Dye **3** exhibited absorption maxima ranging from 415 nm to 466 nm in different solvents (Figure S3A). Interestingly, dichloromethane (DCM) caused the most notable red shift. In comparison with dye **1** and **2**, dye **3** showed two distinctive features. First, it exhibited a dramatic reduction in emission in all solvents (Figure S3B), where the emission was strongest in DMSO and weakest in water. Second, it exhibited a large red-shift in all solvents, with the emission maxima ranging from 600 nm to 630 nm. Additionally, some solvents, such as ethyl acetate and toluene, caused a small blue-shift compared with other solvents (Figure 4C).

**Figure 5:**
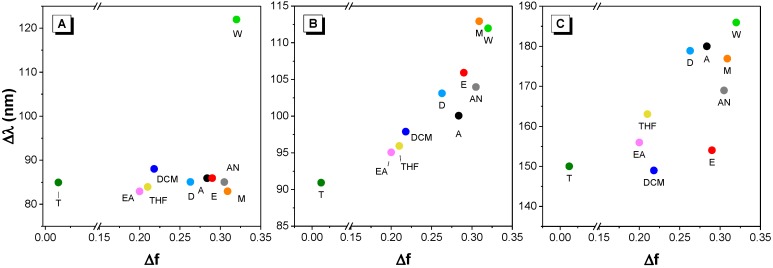
Lippert–Mataga solvatochromism plot of dyes **1** (**A**), **2** (**B**) and **3** (**C**) in different solvents as a function of solvent polarity measured by Lippert-Mataga polarity parameter. A = acetone, AN = acetonitrile, D = DMSO, E = ethanol, EA = ethyl acetate, M = methanol, T = toluene, W = water.

The Stokes shift of all dyes as a function of solvent polarity was graphed in the Lippert–Mataga solvatochromism plot (Figure 5A–C) [26]. It can be observed that, in general, the Stokes shifts of dyes **2** and **3** are mostly proportional to the solvent polarity, suggesting both dyes possess positive solvatochromism, whereas the Stokes shift remained almost unchanged in different solvents with the exception of dye **1** in water.

#### 2.2.3. Effect of Viscosity

**Figure 6:**
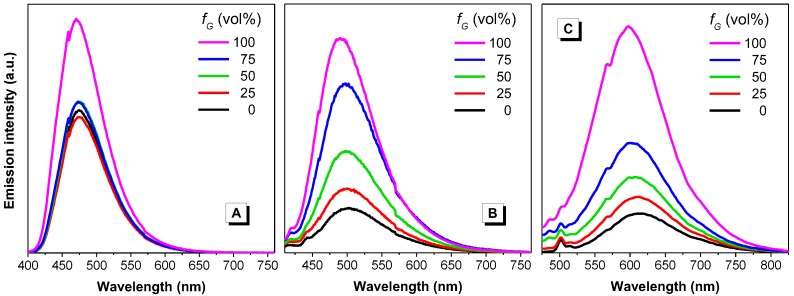
Emission spectra of dyes **1** (**A**), **2** (**B**) and **3** (**C**) in ethylene glycol/glycerol mixtures, where fG indicates glycerol fraction. Concentration = 10 µM. Excitation wavelength: 388, 390 and 437 nm, respectively. Note: in panel C, the peak at approx. 500 nm corresponds to the Raman scattering of the solvent.

We attempted to assess whether our dyes behaved like AIE-active species using viscous solvents, which were expected to restrict the intramolecular motions of the dye molecules, thus enhancing their emission intensity [5,27]. Fluorescence measurements were performed on a series of dye solutions in ethylene glycol/glycerol mixtures, both of which have similar polarity with glycerol being the viscosity enhancer (Figure 6). It was observed that the emission of dye **1** remained similar until the media turned highly viscous (glycerol fraction > 75%), while dyes **2** and **3** showed progressively enhanced emission with the increase of glycerol fraction. It is speculated that, although all these dyes contain rotatable moieties, the strong dipole moment within dyes **2** and **3** can make them more vulnerable to viscosity changes [12].

#### 2.2.4. Effect of Concentration and Aggregation

**Figure 7:**
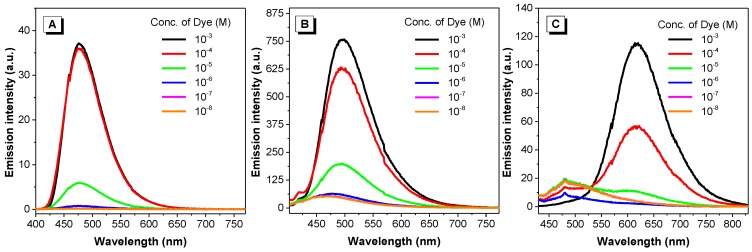
Emission spectra of dyes **1** (**A**), **2** (**B**) and **3** (**C**) at various concentrations in DMSO. Excitation wavelength: 390, 392 and 420 nm, respectively.

We then attempted to further verify the AIE properties of the dyes by inducing aggregation with increased concentration and reduced solubilization. We first took photos of their solubilized and aggregated states under UV light. As illustrated in Figure S4, all dyes are only weakly emissive when molecularly solubilized in acetone, a solvent with strong dissolving power for organic molecules. However, intense fluorescence was observed for all dyes in their solid state, where their concentration is maximized. We then performed serial dilutions to all dyes (solutions in DMSO) and measured their emission intensity at those resulting concentrations (Figure 7). Basically, all dyes were non-emissive or only weakly emissive at concentrations below 10^−5^ M, and then became strongly fluorescent at higher concentrations. Since aggregation is more likely to occur at higher concentrations, some conventional dyes such as pyrene experience fluorescence quenching at higher concentrations. The fluorescence enhancement of these dyes at higher concentrations suggested that all dyes behaved like AIE-active compounds.

**Figure 8:**
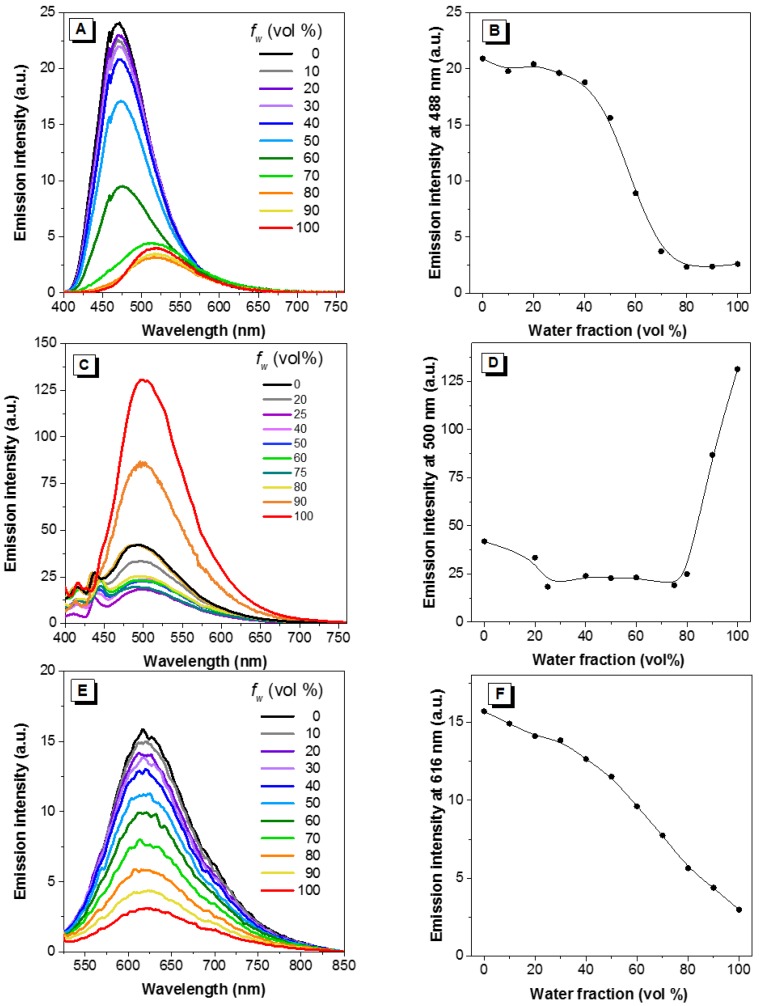
Emission intensity of dyes **1** (**A**,**B**), **2** (**C**,**D**) and **3** (**E**,**F**) in methanol/water mixtures with different water fractions. Concentration = 10 µM. f_w_ and vol% = water fraction. λ_ex_: 385, 387 and 437 nm respectively.

The AIE properties of all dyes were then verified in a series of methanol/water mixtures. Note that these two solvents possess relatively close polarity so as to minimize the influence of solvent polarity. We speculated that dyes would become progressively emissive with increasing water fraction. Strikingly, dye **1** exhibited similar emission until the water fraction increased up to 70%, and then a sharp reduction in intensity was observed at around 80%. A red shift was also observed at 90% and 100% water (to 500 nm and 520 nm), respectively (Figure 8A,B). Similarly, addition of water did not intensify the emission of the charged compound dye **3** (Figure 8E,F). On the other hand, dye **2** showed similar behavior as other AIE-active dyes: it remained weakly emissive until the water percentage increased up to 80%, thereafter showing a sharp increase in intensity (Figure 8C,D).

To search for an explanation for the inconsistency observed with dyes **1** and **3**, dynamic light scattering (DLS) was performed to assess the particle sizes of dyes dispersed in water (Figure S5). The plot showed that the dispersion of dye **1** formed nanoaggregates with average diameter of 165 nm, whereas that of dye **2** was averaged at 523 nm. Reliable particle size data could not be obtained for dye **3**, most likely because aggregation was not formed owing to the positive charge. The influence of particle sizes will be discussed in more detail later.

#### 2.2.5. Mechanistic Understanding

**Figure 9:**
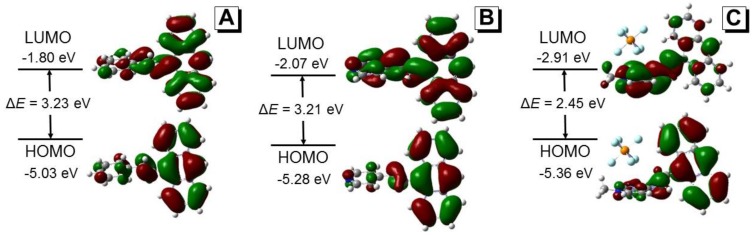
Molecular orbital amplitude plots and the HOMO/LUMO energy levels of **1** (**A**); **2** (**B**) and **3** (**C**) calculated using the B3LYP/6-31G basis set.

Absorption and emission spectra revealed a significant bathochromic shift of dye **3** in solution state when compared to dyes **1** and **2** (Figure 3). To gain insight into the structure–property relationships of these 9-vinylanthracene derivatives, we performed density functional theory (DFT) calculations in Gaussian using the B3LYP/6-31G basis set to obtain the optimized molecular structures and the energy levels of the frontier molecular orbitals (Figure 9). From the optimized structures, the dihedral angles between the anthracene ring and the central C=C double bond were calculated to be 50.4, 49.4 and 44.8 degrees for dyes **1**–**3**, respectively. The smallest dihedral angle in dye **3** suggests that the conjugation of dye **3** is the best among these three dyes and thus explains the observed red-shift in the absorption spectrum. Meanwhile, the band gaps between the highest occupied molecular orbitals (HOMO) and the lowest unoccupied molecular orbitals (LUMO) of dyes **1**–**3** were calculated to be 3.23, 3.21, and 2.45 eV, respectively, which are consistent with our experimental results showing the absorption maxima of 390, 392 and 429 nm, respectively (Figure 3).

**Figure 10:**
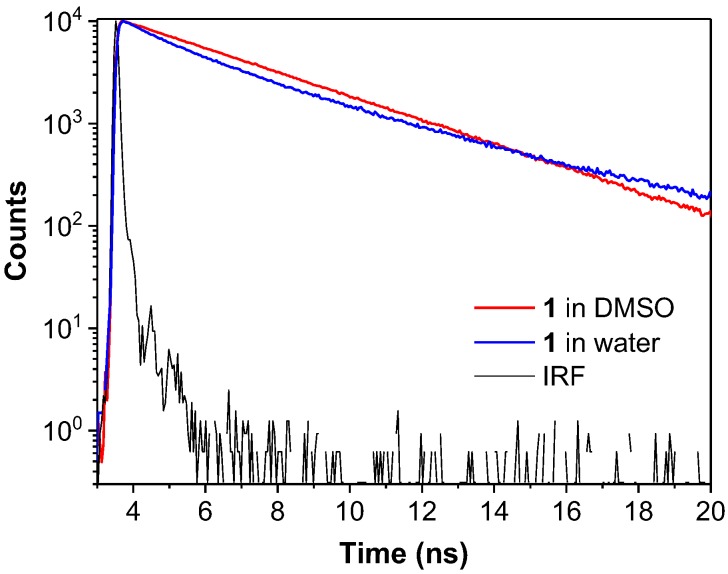
Fluorescence decay profile of dye **1** in DMSO (red) and water (blue). The black line shows the instrument response function (IRF) of the measurement system. Dye concentration: 10 μM. λ_ex_: 400 nm. λ_em_: 480 nm for DMSO and 520 nm for water.

The electron clouds of the HOMO and LUMO of dye **1** are both mostly distributed on the anthracene rings and the central C=C double bond, indicating that intramolecular charge transfer (ICT) processes are unlikely in this molecule [28]. While the HOMO of both dyes **2** and **3** are located on the vinylanthracene moiety, as for dye **1**, the LUMO of dye **2** has a large portion of electron density located at the pyridine unit while the LUMO of dye **3** is mostly located at the pyridinium unit (Figure 9). The difference in the spatial distribution of the HOMOs and LUMOs indicates that the ICT process is feasible in both these dyes. For dye **3**, the distinct electron distribution of the HOMO and LUMO suggests a strong push-pull effect with the vinylanthracene as electron donor and the pyridinium as acceptor [29]. This is in line with our observation of the solvatochromism behaviour of both dyes **2** and **3** in solvents with different polarity whereas dye **1** is almost inert to the change of solvent polarity (Figure 4 and Figure 5). The red shift observed in the emission spectrum of dye **1** in water (Figure 4A) could be attributed to strong intermolecular interactions when the molecules cluster to form dense nanoaggregates (Figure S5A). To further understand the mechanism, we performed time-resolved fluorescence measurements (Figure 10 and Table S1). The multi-exponential decay of dye **1** in water indicates the heterogeneity of aggregates. However, no direct evidence of excimer formation can be found from the fluorescence decay profile.

On the other hand, the large dihedral angles as shown in the optimized structure between the vinylanthracene and the terminal aromatic rings suggest that none of the dyes is coplanar. Because of the steric hindrance, the anthracene ring twists out of the central plane. Thus, the conjugation between anthracene and the central C=C bond is weak, which allows the intramolecular motions of the C-C bond upon excitation [5]. Such intramolecular motions would serve as a non-radiative decay channel to deactivate the excited species. As long as such intramolecular motions are restricted, the fluorescence intensity of the dyes can be enhanced. In viscous medium, such as glycerol, the intramolecular motions would be hampered and thus more excited species can go through the radiative decay pathway. Therefore, with the increase of glycerol fraction, the fluorescence of dyes **1**–**3** is increased (Figure 6).

Conventional ACQ dyes such as pyrene suffer from concentration-caused quenching [5]. Although when the concentration of pyrene is increased from 10^−6^ to 10^−4^ M, the fluorescence is enhanced, further increasing its concentration results in a decrease of the emission. For dyes **1**–**3**, increasing the concentration from 10^−8^ to 10^−3^ M monotonically intensifies the fluorescence intensity of the dyes (Figure 7), as occurs with most AIE luminogens. Increasing the dye concentration also led to the aggregation of the dyes, which, in turn, restricts the intramolecular motions of the dye molecules. In the solid state, the intramolecular motions are hampered greatly and, as observed, all the dyes (**1**–**3**) emit strong fluorescence (Figure S4). Usually increasing the water fraction in solvent mixtures can induce aggregation of hydrophobic dyes; for AIE-active dyes, the fluorescence would increase along with the increase of water fraction. Dye **2** conformed with the behavior of most AIE dyes: its fluorescence remained low when the water fraction in methanol/water mixture is lower than 85% and increased dramatically when the water fraction reaches 99% where nanoaggregates form (Figure 8 and Figure S5). However, for dyes **1** and **3**, increasing the water fraction does not increase their fluorescence. For dye **1**, although nanoaggregates formed at a high water fraction, the fluorescence was weaker than in methanol. A red-shift in the spectrum implies strong intermolecular interactions, predominantly on the anthracene moiety, in the high water fraction and weakened the fluorescence. For dye **3**, due to its amphiphilic property, addition of water could not induce the aggregate formation of the dye molecules and we thus were unable to see the increase of its fluorescence.

#### 2.2.6. Comparison with Other Literature Structural Analogs

The basic spectroscopic and photophysical properties of dyes **1**–**3** are compared with those from representative literature and commercial analogs reported previously (Table 1, structures shown in Figure S7). Entries **4**, **5** and **6** are examples of 9-position mono-functionalised anthracene derivatives. It can be observed that adding extra bulky conjugations to the phenyl moiety of dye **1** (as in entries **4** and **5**) caused red-shift in the emission spectra. Marked red-shift in both absorption and emission can occur after converting the pyridine moiety into a charged pyridinium (**2** vs. **3** and **6**). Considering the listed 9,10-bifunctionalised anthracene derivatives (**7**–**15**), it appears that they generally exhibit absorption and emission maxima at longer wavelength in comparison to their mono-functionalised counterparts (**7** vs. **1** and **15** vs. **2**). Electron-donating and weak electron-withdrawing groups attached to the phenyl moieties of **7** do not incur significant changes to the spectroscopic profiles (**8**–**13**); however, nitrile groups as a strong electron withdrawer can cause large red-shift in the emission spectra (**14**). Lastly, most dyes listed show a very low quantum yield when molecularly solubilised, and this typical AIE phenomenon is consistent with the observations in dyes **2** and **3**.

### 2.3. Applications

#### 2.3.1. Protonation Effect

**Figure 11:**
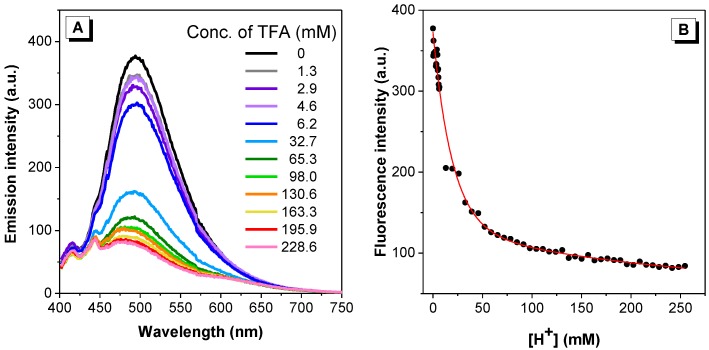
(**A**) Emission spectra of dye **2** in DMSO with increasing concentration of TFA. Dye concentration = 20 µM; λ_ex:_ 392 nm; (**B**) emission intensity of dye **2** vs. proton concentration.

In comparison with commonly used electrochemical pH sensors, optical pH sensors possess a number of advantages including contactless measurement, ease of miniaturization and possibility of performing concurrent imaging [38]. Based on the well-established understanding that the degree of protonation may impose significant changes to the absorption/emission [39,40,41], we selected the pyridine-bearing dye **2** to assess its capability in sensing pH alternation in solutions. Solutions varying in proton concentration were prepared using trifluoroacetic acid (TFA) as a proton source. As shown in Figure 11, dye **2** was found to be strongly emissive in neutral and moderately acidic environments, and then exhibited a sharp reduction in emission up to a 50 mM proton concentration. Similar results were observed with dye **2** in aqueous media (Figure S6). An exponential relationship between the emission intensity and proton concentration was established, suggesting that dye **2** may be a suitable optical pH sensor for conditions ranging from neutral to strong acidic environments.

#### 2.3.2. Protein Detection

**Figure 12:**
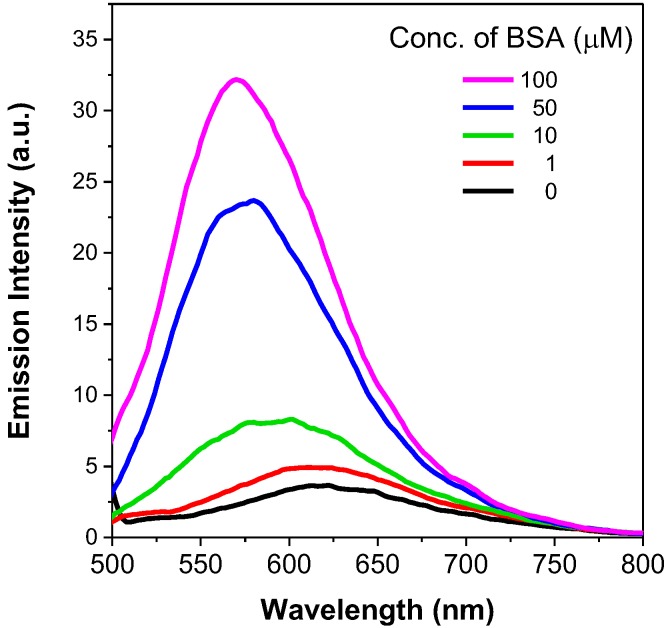
Fluorescence emission spectra of dye **3** with various concentrations of BSA in phosphate-buffered saline (PBS) excited at 420 nm.

Fluorometric methods for protein detection and quantification are becoming increasingly used in research fields, owing to their satisfactory sensitivity, low background noise and wide dynamic ranges. Protein binding onto AIE-active dyes has been shown to enhance emission intensity [42]. In this work, we selected dye **3** for investigation as its positive charge enabled comparatively higher aqueous solubility than dyes **1** and **2**. Its interaction with a model protein, bovine serum albumin (BSA), was analysed in a pH 7.0 phosphate buffer. As illustrated in Figure 12, dye **3** was only weakly emissive in buffer solution in the absence of BSA. Addition of BSA progressively intensified its fluorescence emission, with the emission maxima shifting from 620 nm to 575 nm. As BSA contains hydrophobic binding pockets in its native folded structure, we speculated that the intramolecular rotation of BSA-bound dye **3** had been hindered, hence emitting in a manner similar to that observed in an aggregated state. Meanwhile, the blue-shift of the spectra indicated the transition from a hydrophilic to hydrophobic environment of the dye molecules. These observations again proved that dye **3** is AIE-active and therefore is a valuable candidate for protein fluorescent detection and quantification in proteomics.

#### 2.3.3. Cell Imaging

**Figure 13:**
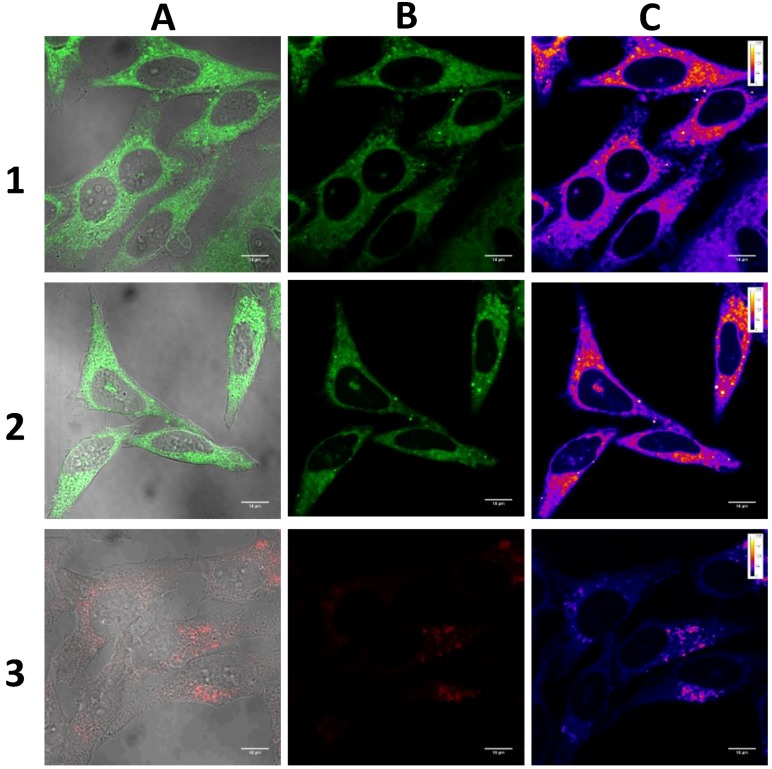
Confocal fluorescence microscopy image of HeLa cells stained with dyes **1**, **2** and **3** (10 µM) for 30 min. (**A**) Merged image of the fluorescence and bright-field images; (**B**) fluorescence image under excitation of 405 nm laser (2.0% for dyes **1** and **2**, 6.0% for dye **3**); (**C**) the intensity of the signal was visualised using the “Fire” lookup table (LUT) colour scheme in Image J2. Linear mapping of pixel values in the display range indicated by the calibration bar (0–255 a.u.). Scale bar: 15 µm.

All dyes were assessed to determine their potential as luminescent probes for bioimaging. We stained live HeLa cells with dyes **1**, **2** and **3** and fixed the cells before conducting confocal microscopy bioimaging. The results indicated that all the dyes were capable of entering the cytoplasm, but only **1** and **2** displayed some nuclear penetration (Figure 13). This could be largely due to the anthracene group, which is able to be taken up by lysosomes and then released into the nucleus along with lysosomal deoxyribonuclease upon prolonged exposure to light [43]. Additionally, there was little or no background fluorescence when stained with these three dyes. This further validated their bioimaging potential as dyes **1**, **2** and **3** were able to enter cells whilst exhibiting no off-target fluorescence. To visualise the intensity of their luminescence, we then used the Fire lookup table (LUT) colour scheme on the Fiji software (ImageJ2, LOCI, Madison, WI, USA) to compare their linearized pixel values in the display range (0–255 a.u.). Results revealed that the luminescence intensity of dyes **1** and **2** were relatively similar, with dye **3** exhibiting the least intense luminescence (Figure 13C). Moreover, luminescence was only visible when dye **3** was excited with a higher laser power (6.0%) compared with **1** and **2**, which were both excited at a lower laser power (2.0%).

Compared with dyes **1** and **2**, dye **3** exhibited weaker fluorescence in the cells. We postulated that the main reason is the hydrophilicity of the dye, which makes it difficult to get through the hydrophobic interior of the plasma membrane [44]. Furthermore, the low fluorescence quantum yield of **3** could be another reason for its weak signal from the cells (Figure S3B). Its overall positive charge and amphiphilic nature may hinder its ability to form aggregates and enhance its luminescence. In addition, in contrast to traditional AIE dyes such as TPE, all three compounds experienced significant and rapid photobleaching upon prolonged laser exposure, most likely due to the oxidation of the anthracene moiety.

## 3. Experimental Section

### 3.1. Materials and General Information

9-Bromoanthracene, styrene and 4-vinylpyridine were purchased from Alfa Aesar (Haverhill, MA, USA). Pd(OAc)_2_, PPh_3_, KPF_6_, MgSO_4_, trifluoroacetic acid (TFA) and bovine serum albumin (BSA) were purchased from Sigma Aldrich (St. Louis, MO, USA). K_3_PO_4_ and K_2_CO_3_ were purchased from Chem-supply (Adelaide, SA, Australia). CH_3_I and all solvents were purchased from Merck (Darmstadt, Germany). All chemicals were used without further purification.

^1^H-NMR and ^13^C-NMR spectra were recorded at 400 MHz using a 400 MHz Bruker AV3HD-400 spectrometer (Billerica, MA, USA) at 298 K. UV-Visible absorption spectra were recorded at room temperature on a Cary 300 UV-visible spectrophotometer (Agilent Technologies, Inc., Santa Clara, CA, USA) equipped with a 1.0 cm quartz cell. Fluorescence emission and excitation spectra were recorded on a Cary Eclipse Fluorescence Spectrophotometer (Agilent Technologies, Inc., Santa Clara, CA, USA) and used 1.0 cm quartz cells. Particle sizes were measured by dynamic light scattering using Zetasizer Nano S90 (Malvern Instruments Ltd., Malvern, UK). Data were plotted by using Origin 2016 (OriginLab Corp., Northampton, MA, USA).

### 3.2. Synthesis and Characterization

*(E)-9-Styrylanthracene* (**1**). The synthetic route towards (**1**) was adopted from the strategies previously described by Yao et al. [25]. To an oven-dried 25 mL round-bottom flask were added 9-bromoanthracene (0.3 g, 1.17 mmol), styrene (0.12 g, 1.17 mmol), K_3_PO_4_ (0.75 g, 3.51 mmol), Pd(OAc)_2_ (0.01 g, 0.06 mmol) and dry DMA (10 mL). The mixture was degassed by three freeze-pump-thaw cycles, and then stirred under N_2_ at 110 °C for 24 h. The mixture was poured into water and extracted with DCM. The combined organic layer was washed with brine, dried over anhydrous MgSO_4_ and concentrated in vacuo. Water (6 × 7 mL) was added to the resulting mixture to extract DMA. The crude product was dried in vacuo to give a bright yellow solid (100 mg, isolated yield = 30%). ^1^H-NMR (400 MHz, CDCl_3_): δ 6.97 (d, 1H), 7.35–7.41 (m, 1H), 7.44–7.52 (m, 6H), 7.67–7.73 (d, 2H), 7.93 (d, 1H), 7.99–8.06 (m, 2H), 8.34–8.44 (m, 3H). ^13^C-NMR (400 MHz, CDCl_3_): δ 124.91, 125.21, 125.49, 126.05, 126.49, 126.63, 128.04, 128.72, 128.87, 129.77, 131.54, 132.79, 137.35.


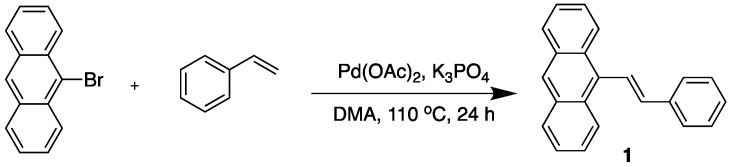


*(E)-4-(2-(Anthracen-9-yl)vinyl)pyridine* (**2**). The synthetic route towards (**2**) was adopted from the strategies previously described by Shih et al. [22]. To an oven-dried 50 mL Schlenk tube were added 9-bromoanthracene (1.0 g, 3.9 mmol), K_2_CO_3_ (1.6 g, 11.6 mmol), PPh_3_ (153 mg, 0.6 mmol) and Pd(OAc)_2_ (44 mg, 0.2 mmol). After the system was sealed and evacuated, dry DMF (10 mL) and 4-vinylpyridine (613 mg, 630 μL, 5.8 mmol) were injected. The mixture was degassed by three freeze-pump-thaw cycles, and then stirred under N_2_ at 110 °C for 24 h. The reaction mixture was poured into a LiCl solution (5% in 100 mL H_2_O), and the suspension was extracted with EtOAc. The combined organic layer was washed by brine, dried over anhydrous MgSO_4_ and concentrated in vacuo. The crude product was purified by column chromatography (PET:EtOAc, 4:1 then 1:1) and a bright yellow powder (250 mg, isolated yield = 23%) was collected. ^1^H-NMR (400 MHz, CDCl_3_): δ 6.92 (d, 1H), 7.48–7.57 (m, 6H), 8.02–8.07 (m, 2H), 8.16 (dd, 1H), 8.26–8.32 (m, 2H), 8.46 (s, 1H), 8.71 (s, 2H). ^13^C-NMR (400 MHz, CDCl_3_): δ 121.03, 125.33, 125.52, 125.96, 127.36, 128.87, 129.61, 130.00, 131.26, 131.43, 134.78, 144.56, 150.24. ESI-MS: *m*/*z* 282.06, calcd. 281.36.


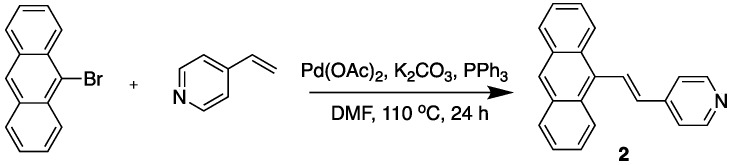


*(E)-4-(2-(Anthracen-9-yl)vinyl)-1-methylpyridin-1-ium hexafluorophosphate* (**3**). To a 100 mL round-bottom flask were added compound **2** (230 mg, 0.8 mmol), acetone (5 mL) and iodomethane (2 mL). The mixture was refluxed at 50 °C for 1 hour. The precipitate was collected by filtration, washed by acetone and air dried. This intermediate compound (**3′**) was collected as a pale orange powder in quantitative yield. ^1^H-NMR (400 MHz, DMSO-*d*_6_): δ 4.34 (s, 3H), 7.34 (d, 1H), 7.55–7.67 (m, 4H), 8.15–8.22 (m, 2H), 8.35–8.41 (m, 2H), 8.51 (d, 2H), 8.72 (s, 1H), 8.91–9.03 (m, 3H). ^13^C-NMR (400 MHz, DMSO-*d*_6_): δ 47.59, 124.63, 125.76, 126.14, 127.21, 128.90, 129.41, 129.51, 130.42, 131.41, 132.53, 137.95, 145.75, 152.29.


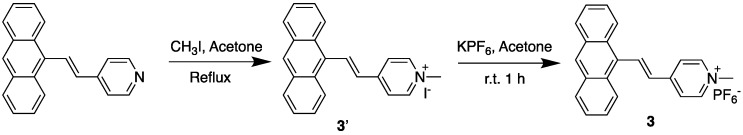


To a 50 mL round-bottom flask were added **3′** (120 mg, 0.3 mmol), acetone (25 mL) and saturated KPF_6_ solution (5 mL). The resulting solution was stirred at room temperature for 1 hour. After the solvent was dried in vacuo, the solid was collected by filtration, washed with H_2_O and dried in the oven. The product was obtained as a deep orange powder (105 mg, isolated yield = 84%). ^1^H-NMR (400 MHz, DMSO-*d*_6_): δ 4.34 (s, 3H), 7.33 (d, 1H), 7.57–7.65 (m, 4H), 8.15–8.20 (m, 2H), 8.35–8.41 (m, 2H), 8.50 (d, 2H), 8.72 (s, 1H), 8.91–9.01 (m, 3H). ^13^C-NMR (400 MHz, DMSO-*d*_6_): δ 47.56, 124.63, 125.74, 126.14, 127.21, 128.91, 129.42, 129.51, 130.42, 131.42, 132.53, 137.94, 145.75, 152.30. ESI-MS: *m*/*z* 296.06 (anthracene-pyridinium), 144.88 (PF_6_^−^), calcd. 296.39 (anthracene-pyridinium), 144.96 (PF_6_^−^).

### 3.3. Sample Preparation for Spectroscopy Measurement

All stock solutions of dyes were prepared in 1 mM in DMSO and kept at room temperature in dark. BSA stock solution was prepared in PBS. The total concentration of all stock solutions was 1 mM. For UV-vis absorption measurement, the background of solvent alone was subtracted. For fluorescence measurement, the excitation and emission slits were fixed at 5 nm for all experiments. Scan speed was set at medium and the curves presented were the average of 3 times measurement in every experiment. The working concentration of dyes was 10 μM unless specified elsewhere.

### 3.4. Quantum Yield Measurements

Absolute quantum yield measurements were performed using a Quanta-Phi HORIBA Scientific 6 in. diameter integrating sphere (HORIBA Scientific, Edison, NJ, USA) at room temperature (22 ± 2 °C) Photoexcitation was with a 450 W arc-xenon lamp and the emission was directed toward a nitrogen cooled Symphony II xenon CCD (Model SII-1LS-256-06, HORIBA Scientific, Edison, NJ, USA) CCD via optical fibers. FluorEssence v3.5 software (HORIBA, Edison, NJ, USA) was used to calculate the quantum yields in a 4-curve analysis mode using the following equation:

$$\Phi p=\frac{\mathrm{Photons}\;\mathrm{out}}{\mathrm{Photons}\;\mathrm{in}}=\frac{(\mathrm{Ec}-\mathrm{Ea})/A}{\mathrm{La}-\mathrm{Lc}}$$

where Ec is the integrated luminescence of the sample resulted by direct excitation, Ea is the integrated luminescence of the blank, La is the integrated excitation from the blank and Lc is the integrated excitation from the sample, and A is the area balance factor obtained from the multiplication of the CCD integration time. Spectral measurements were averaged from at least three replicates.

### 3.5. Particle Size Measurements

As part of the experiment, 20 μL of stock solution of dye **1** and **2** was added in 2 mL Milli-Q water and kept in a 15 mL Falcon tube at room temperature. The total concentration was around 10 µM. The particle size distribution was measured via Zetasizer Nano S90 (Malvern Instruments Ltd., Malvern, UK).

### 3.6. Time-Resolved Fluorescence Measurements

Fluorescence decay measurements were performed by the time-correlated single-photon counting technique using the frequency doubled output (400 nm) of a mode-locked and cavity-dumped Titanium: sapphire laser (Coherent Mira 900f). The repetition rate of the laser pulses was reduced to 5.4 MHz (APE PulseSwitch, Angewandte Physik & Elektronik GmbH, Berlin, Germany). Emission was collected under magic angle conditions, spectrally selected (480 nm or 520 nm) using a Jobin Yvon H20 monochromator (Longjumeau Cédex, France) and detected with a microchannel plate photomultiplier (Eldy EM1-132-1, St. Petersburg, Russia). Data were acquired using a Becker and Hickl SPC-150 TCSPC card (Becker & Hickl GmbH, Berlin, Germany) and SPCM software (version 9.6, Becker & Hickl GmbH, Berlin, German), and analysed using the FAST (Fluorescence Analysis Software Technology) software (version 3.1, Edinburgh Instruments, Livingston, UK).

### 3.7. Cell Culture and Imaging

#### 3.7.1. Cell Culture

HeLa (human cervical cancer) cell line was purchased from American Type Culture Collection (ATCC^®^ CCL2™) and used within 40 passages of purchase. The cells were cultured in Dulbecco’s modified Eagles Medium (DMEM) (HyClone^®^) with 10% fetal calf serum (FCS) (Thermo Fisher Scientific Inc., Waltham, MA, USA) and Penicillin-Streptomycin (P/S) (Thermo Fisher Scientific Inc.), and incubated at 5% CO_2_, 37 °C. 

Dyes **1**, **2** and **3** were stored as stock solutions at 1 mM in dimethyl sulfoxide (DMSO), purchased from Sigma-Aldrich Co.

#### 3.7.2. Cell Staining and Confocal Imaging

Pre-sterilized µ-Slide 8 Well (Ibidi^®^) slides were coated with 1 mL/25 cm^2^ of Poly-l-Lysine (Sigma-Aldrich Co., St. Louis, MO, USA) for 2 h prior to cell seeding to promote cell adherence. Cells were then seeded at a density of 7 × 10^5^ cells per well and incubated in cell culture medium for 48 h at 5% CO_2_ and 37 °C. Afterwards, cells were first fixed in 4% paraformaldehyde solution (PFA) in Phosphate Buffered Saline (PBS) (HyClone^®^) at 37 °C for 5 min to further promote cell adherence. Prior to cell staining, stock solutions of **1**, **2** and **3** 1 mM in DMSO were diluted in DMEM (HyClone^®^) without FCS and PBS to a working concentration of 50 µM.

Following washing with PBS, the cells were then stained with 50 µM of dyes **1**, **2** or **3** at room temperature for 30 min. After a further wash with PBS, the stained and fixed cells were then stored in cold PBS. 

Imaging was performed on the slides using the LSM 780 confocal microscope (Zeiss, Jena, Germany), equipped with an AxioObserver Z1 inverted microscope, 63× oil immersion objective lens (NA 1.4), 405 nm diode laser (30 mW), and a multi-anode PMT (32 elements). Luminescence spectrum was registered from single confocal sections (pinhole set to 1 Airy unit), in 427–529 nm range, using 405 nm (dye **1** and **2**—2.0% power, dye **3**—6.0% power). Images were processed using the free Fiji software (ImageJ2, LOCI, Madison, WI, USA).

## 4. Conclusions

In summary, we have synthesized three fluorescent dyes derived from the 9-position functionalized anthracene moieties using a simple and robust Pd-catalyzed Heck reaction. We then demonstrated their enhanced emission in the aggregated state using standard fluorometric techniques, and established their structure–property relationships using DFT calculations. Additionally, the results of further experiments described here suggest the potential of these dyes in such applications as pH sensing, protein detection and quantification, and, for the first time, in fluorescence live cell imaging.

## Figures and Tables

**Table 1:** Spectroscopic and photophysical properties of Dyes **1**–**3** compared to previously reported analogs. ^[a]^

Entry	Compounds	Absorbance λ_max_/nm	PL in Solution λ_max_/nm	Φ_PL_ (%) in Solution	Ref.
**1**	dye **1** ^[b]^	390	475	60	This work
**2**	dye **2** ^[b]^	392	495	3	
**3**	dye **3** ^[b]^	385, 420	618	<1	
**4**	*t*-2-ANE ^[c],^*	387	490	68	[30]
**5**	1,4-ASB ^[d],^*	342, 390	510	15	[31]
**6**	“Chromophore 9” ^[d]^	442	N/A	N/A	[32]
**7**	DSA ^[d],^*	304, 414	555	0.4	[33]
**8**	BMOSA ^[d],^*	307, 415	550	0.7	
**9**	B-4-BOSA ^[d]^	307, 417	538	0.5	
**10**	B-2-BOSA ^[d]^	313, 415	567	2.3	
**11**	9,10-MADSA ^[d]^	~315, ~422	~550	0.5–0.8	[34]
**12**	9,10-PADSA ^[d]^	~315, ~422	~550	0.5–0.8	
**13**	BCSA ^[e],^*	403	576	N/A	[35]
**14**	CNDSA ^[d]^	418	625	25	[36]
**15**	BP4VA ^[d],^*	410	584	N/A	[37]

^[a]^ The chemical structures of all the compounds are shown in Supplementary Information. ^[b]^ solvent = DMSO; ^[c]^ solvent = acetonitrile; ^[d]^ solvent = THF; ^[e]^ solvent = DCM. * = commercially available.

## Supplementary Materials

Supplementary materials are available online.
