# Supplementary material for: 9-Vinylanthracene Based Fluorogens: Synthesis, Structure-Property Relationships and Applications
Source: Molecules. 2017 Dec 4;22(12):2148. doi: 10.3390/molecules22122148 (PMC6149741; doi:10.3390/molecules22122148)
Supplement: Supplementary file 1 [file molecules-22-02148-s001.pdf]

9-Vinylanthracene Based Fluorogens: Synthesis, Structure-Property Relationships and Applications

Supplementary Information

Mengjie Liu [1,†], Sawaros Onchaiya [1,†,‡], Lewis Yi Fong Tan [1], Mohammad A. Haghighatbin [1], Tracey Luu [1], Tze Cin Owyong [1,2], Roozbeh Hushiarian [1], Conor F. Hogan [1], Trevor A. Smith [2] and Yuning Hong [1,*]

[1] Department of Chemistry and Physics, La Trobe Institute for Molecular Science, La Trobe University, Melbourne, VIC 3086 Australia; M.Liu4@latrobe.edu.au (M.L.); sawaros.onc@student.mahidol.ac.th (S.O.); 18768730@students.latrobe.edu.au (L.Y.F.T); M.Haghighatbin@latrobe.edu.au (M.A.H.); traceyl@student.unimelb.edu.au (T.L.); towyong@student.unimelb.edu.au (T.C.O.); R.Hushiarian@latrobe.edu.au (R.H.); C.Hogan@latrobe.edu.au (C.F.H.)

[2] School of Chemistry, The University of Melbourne, Parkville, VIC 3010 Australia; trevoras@unimelb.edu.au

* Correspondence: Y.Hong@latrobe.edu.au; Tel.: +61-3-9479-2995

† Co-first authorship

‡ Current address: Department of Chemistry, Faculty of Science, Mahidol University, Bangkok, 10400, Thailand

Table of Contents

Figure S1. (**A**) Normalized absorption spectra of dye **1** in different solvents; (**B**) Emission spectra of dye **1** in different solvents. 2

Figure S2. (**A**) Normalized absorption spectra of dye **2** in different solvents; (**B**) Emission spectra of dye **2** in different solvents. 2

Figure S3. (**A**) Normalized absorption spectra of dye **3** in different solvents; (**B**) Emission spectra of dye **3** in different solvents. 3

Figure S4. Photographs of dyes **1-3** taken under UV radiation (366 nm) from a hand-held UV lamp. Left: dye **1-3** in solid state; right: dyes **1-3** dissolved in acetone compared to their solid state. 3

Figure S5. Dynamic Light Scattering analysis of (**A**) dye **1** and (**B**) dye **2** particles via the Zetasizer Ver 7.01 program. 3

Figure S6. Response of dye **2** in water with increasing concentration of TFA. 4

Figure S7. Structures of anthracene analogues listed in Table 1. 4-5

Table S1. Fluorescence decay parameters of dye **1**. 5

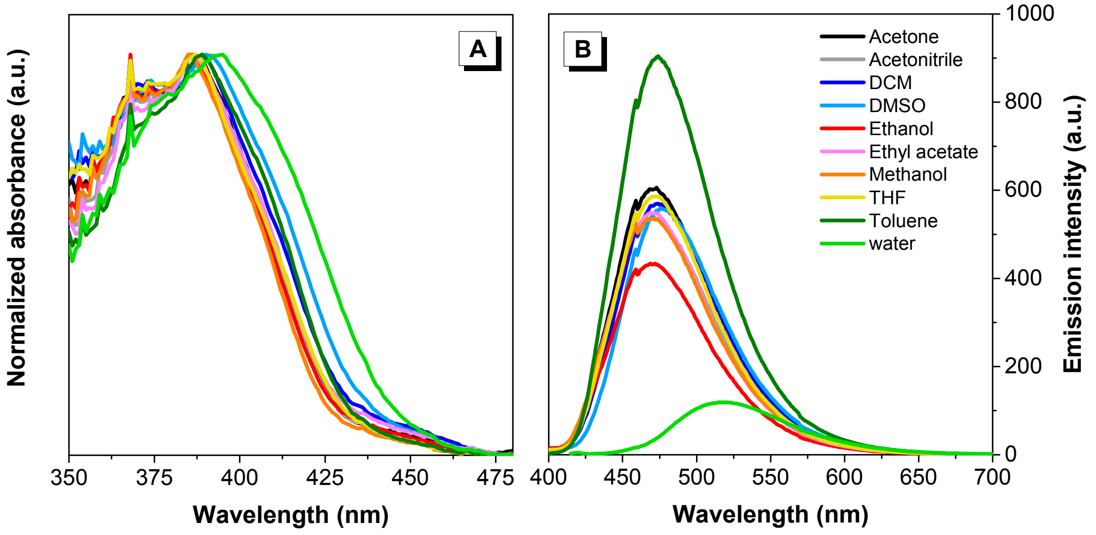

Figure S1. (**A**) normalized absorption spectra of dye **1** in different solvents; (**B**) emission spectra of dye **1** in different solvents. Concentration = 10 μM; λ_{ex}: 385 - 395 nm.

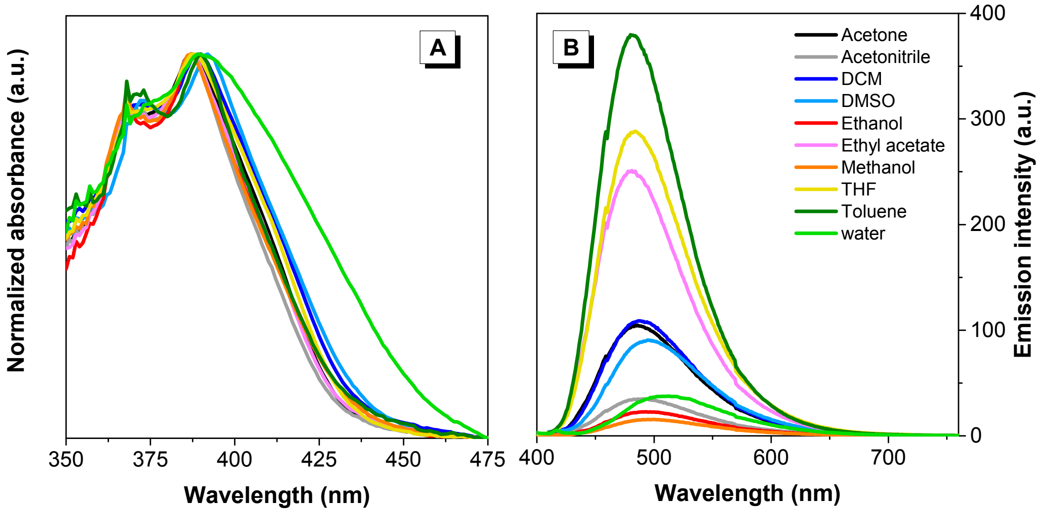

Figure S2. (**A**) normalized absorption spectra of dye **2** in different solvents; (**B**) emission spectra of dye **2** in different solvents. Concentration = 10 μM; λ_{ex}: 387 - 392 nm.

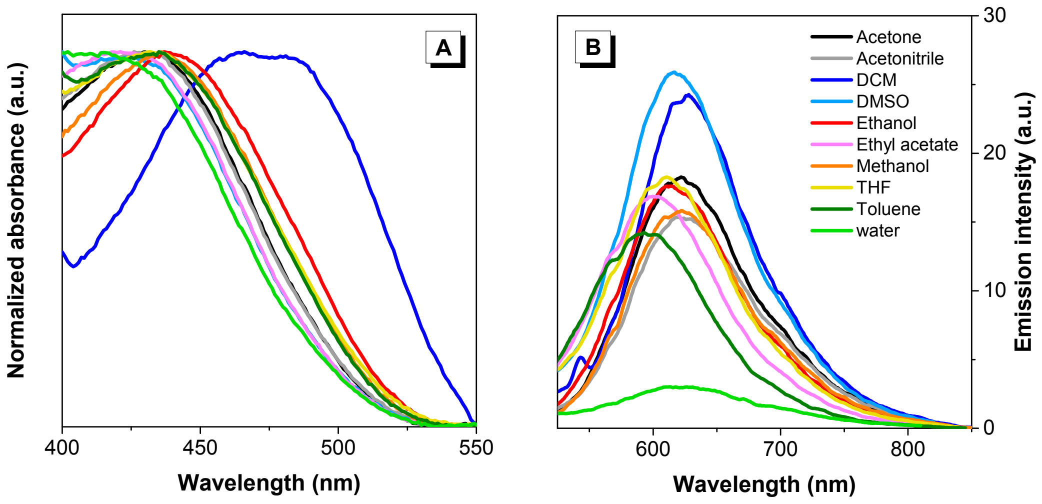

Figure S3. (**A**) normalized absorption spectra of dye **3** in different solvents; (**B**) emission spectra of dye **3** in different solvents. Concentration = 10 μM; λ_{ex}: 415 - 466 nm.

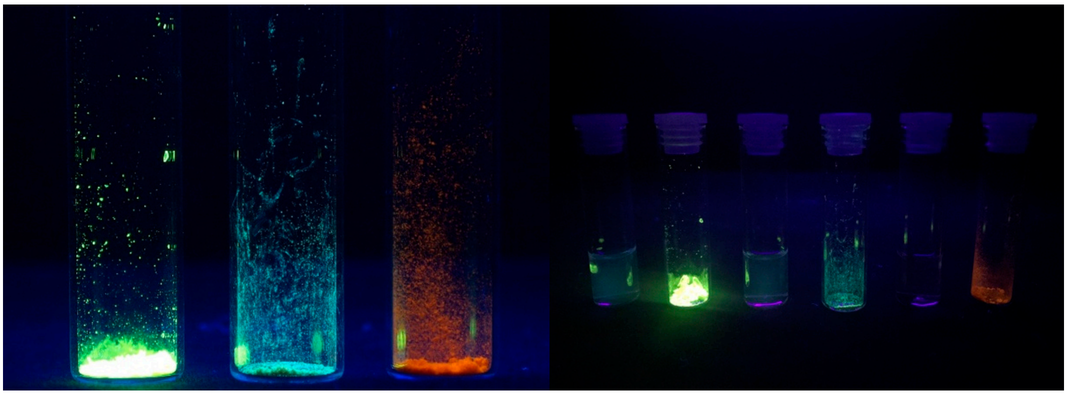

Figure S4. Photographs of dyes **1-3** taken under UV radiation (366 nm) from a hand-held UV lamp. Left: dyes **1-3** in solid state; right: dyes **1-3** dissolved in acetone compared to their solid state.

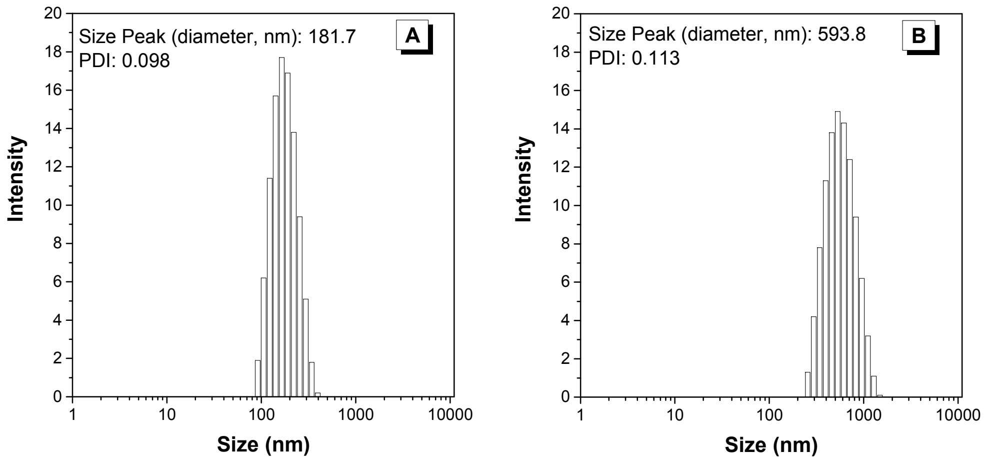

Figure S5. Dynamic Light Scattering analysis of (A) dye **1** and (B) dye **2** particles via the Zetasizer Ver 7.01 program.

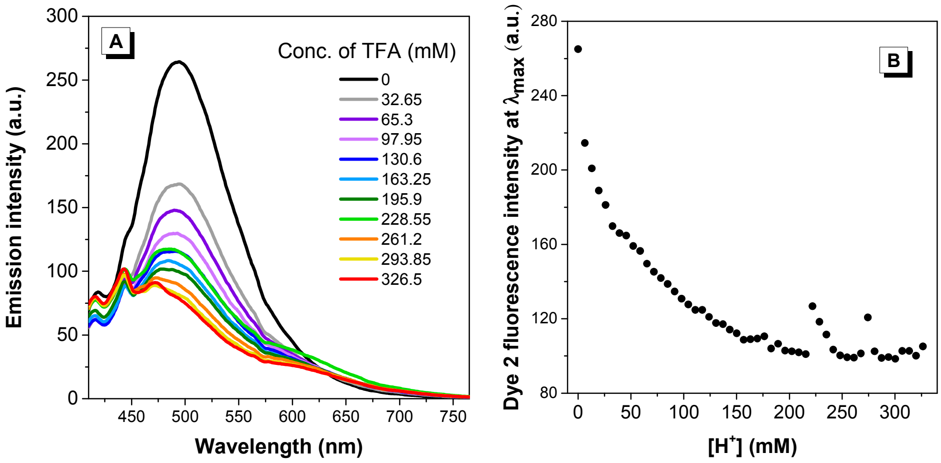

Figure S6. (**A**) emission spectra of dye 2 in water with increasing concentration of TFA. Concentration = 20 μM; λ_{ex}: 392 nm; (**B**) emission intensity of dye **2** at 488 nm vs. proton concentration.

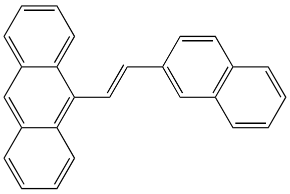

t-2-ANE

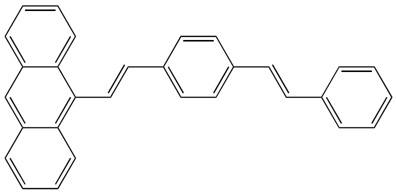

1,4-ASB

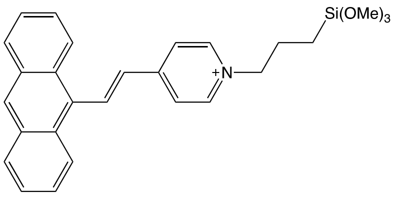

"Chromophore 9"

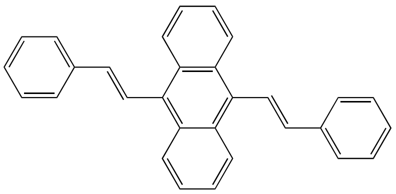

DSA

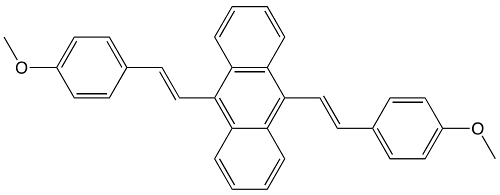

BMOSA

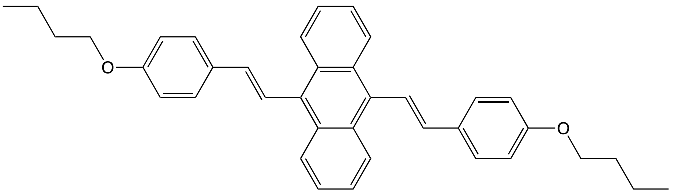

B-4-BOSA

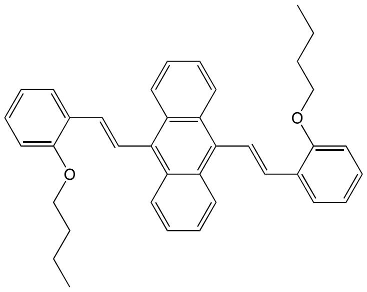

B-2-BOSA

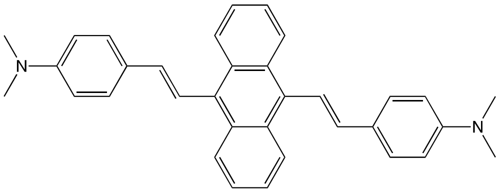

9,10-MADSA

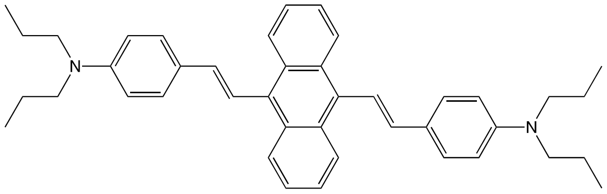

9,10-PADSA

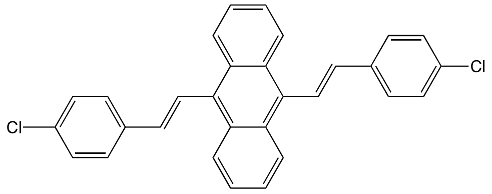

BCSA

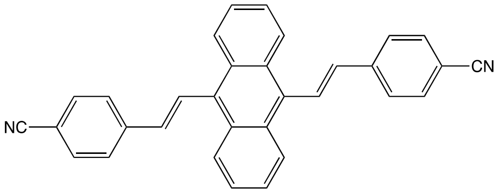

CNDSA

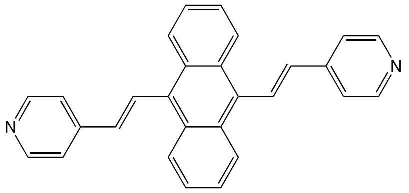

BP4VA

Figure S7. Structures of anthracene analogs listed in Table 1.

Table S1. Fluorescence decay parameters of dye **1**.

Species[a]	A_1[b]	A_2[b]	A_3[b]	τ_1 [ns][b]	τ_2 [ns][b]	τ_3 [ns][b]
Dye **1**/DMSO	100			3.690		
Dye **1**/H_2O	61.5	28.4	10.1	4.849	2.230	1.068

[a] [**1**] = 10μM. [b] Determined from Equation 1, where A and τ are the fractional amount and fluorescence lifetime of different species, respectively.

$$I = A_1 e^{-\frac{t}{\tau 1}} + A_2 e^{-\frac{t}{\tau 2}} + A_3 e^{-\frac{t}{\tau 3}} \quad \text{eq.1}$$
